# All-in-One Photoactivated Inhibition of Butyrylcholinesterase Combined with Luminescence as an Activation and Localization Indicator: Carbon Quantum Dots@Phosphonate Hybrids

**DOI:** 10.3390/nano13172409

**Published:** 2023-08-25

**Authors:** Gulia Bikbaeva, Anna Pilip, Anastasia Egorova, Ilya Kolesnikov, Dmitrii Pankin, Kirill Laptinskiy, Alexey Vervald, Tatiana Dolenko, Gerd Leuchs, Alina Manshina

**Affiliations:** 1 Institute of Chemistry, St. Petersburg State University, St. Petersburg 199034, Russia; st086467@student.spbu.ru; 2 Center for Optical and Laser Materials Research, St. Petersburg State University, St. Petersburg 199034, Russia; ie.kolesnikov@gmail.com (I.K.);; 3 St. Petersburg Federal Research Center of the Russian Academy of Sciences (SPC RAS), Scientific Research Centre for Ecological Safety of the Russian Academy of Sciences, St. Petersburg 197110, Russia; 4 World-Class Laboratory, St. Petersburg State Technological Institute (Technical University), St. Petersburg 190013, Russia; 5 D.V. Skobeltsyn Institute of Nuclear Physics, M.V. Lomonosov Moscow State University, Moscow 119991, Russia; laptinskiy@physics.msu.ru (K.L.);; 6 Max Planck Institute for the Science of Light, 91058 Erlangen, Germany

**Keywords:** multifunctional materials, carbon quantum dots, butyrylcholinesterase inhibition, luminescence, photoactivation of bioactivity, photopharmacology

## Abstract

Photopharmacology is a booming research area requiring a new generation of agents possessing simultaneous functions of photoswitching and pharmacophore. It is important that any practical implementation of photopharmacology ideally requires spatial control of the medicinal treatment zone. Thus, advances in the study of substances meeting all the listed requirements will lead to breakthrough research in the coming years. In this study, CQDs@phosphonate nanohybrids are presented for the first time and combine biocompatible and nontoxic luminescent carbon quantum dots (CQDs) with photoactive phosphonate enabling inhibition of butyrylcholinesterase (BChE), which is a prognostic marker of numerous diseases. The conjunction of these components in hybrids maintains photoswitching and provides enhancement of BChE inhibition. After laser irradiation with a wavelength of 266 nm, CQDs@phosphonate hybrids demonstrate a drastic increase of butyrylcholinesterase inhibition from 38% up to almost 100% and a simultaneous luminescence decrease. All the listed hybrid properties are demonstrated not only for in vitro experiments but also for complex biological samples, i.e., chicken breast. Thus, the most important achievement is the demonstration of hybrids characterized by a remarkable combination of all-in-one properties important for photopharmacology: (i) bioactivity toward butyrylcholinesterase inhibition, (ii) strong change of inhibition degree as a result of laser irradiation, luminescence as an indicator of (iii) bioactivity state, and of (iv) spatial localization on the surface of a sample.

## 1. Introduction

Photopharmacology is based on the concept of “smart drugs”—substances whose biological activity is controlled, e.g., by light. Photopharmacology targets therapies for particular organs of the body with precisely determined time and duration of treatment. The ultimate goal is switching on or off the bioactivity of a photopharmacological agent with a light beam, which helps avoid off-target toxicity [1,2]. Currently, available specimens of photopharmacological agents are a combination of two components—*pharmacophore* and *photoswitch* or *photoactivator*. A photoswitch is a compound changing structural geometry or conformation as well as chemical properties under optical radiation. As a photoswitch, one primarily uses azobenzenes, quinoline derivatives, diarylethenes, diazocins, or aryl azoindazoles [3,4,5]. Pharmacophore features refer to particular biological or pharmacological interactions with the biological target or substrate. Various antibiotics (*Vancomycin* and *Cephalosporin*) and anti-Alzheimer’s medicine *Takrin* have been demonstrated as a pharmacophore [6,7]. However, pharmacophores often lose their effectiveness as a result of conjugation with photoswitches or photoactivators. Despite the unequivocal promise of the photopharmacological approach, the aforementioned problem of maintaining the medicinal effect during the conjugation of the components remains a challenge. The latter circumstance determines the interest in the development of substances possessing simultaneously biological activity and the ability to change their function under illumination by light. However, it should be noted that, so far, there are only a limited number of works reporting the successful use of this strategy, and most of them are devoted to the creation of such substances for photodynamic cancer therapy [8]. In addition, a limiting factor is the extremely long and laborious procedure of searching for compounds with significantly different biological activity in different conformational states. In this regard, the relevance of the development of compounds with photomodulated bioactivity becomes critical for progress in photopharmacology.

In earlier works, we demonstrated that a particular family of phosphonates has the remarkable property of both biological activity and the ability to change this biological activity value significantly after laser irradiation [9,10]. In vitro experiments have shown the pronounced inhibition towards enzymes of the cholinesterase group and the modification of this inhibition after laser exposure. The field of application of organophosphorus compounds with anticholinesterase action relates to the treatment of a sizeable list of diseases from neurodegenerative disorders, one of which is Alzheimer’s disease, to skin diseases—psoriasis, epithelial cell injuries, vitiligo, atopic dermatitis, etc. [11,12,13,14]. That is why photoactive phosphonates open the path to a new strategy for the treatment of these diseases based on a photopharmacological approach. Our further experiments demonstrated that fullerene@phosphonate hybrids not only retained their biological activity and photoactivation ability under the action of a laser, but also showed an increase in the inhibition difference in diverse conformational states [15].

It is important to note that the principle of photopharmacology is of particular importance for the treatment of “localized” diseases affecting certain organs or body systems. In this regard, progress in the spatiotemporal control of the area of influence of a biologically active substance on organs and tissues would be a significant advance toward the development of “smart drugs”. The problem of visualizing the distribution of a drug in body tissues is solved by creating and implementing hybrid nanomaterials. In such hybrids, the visualization function could be performed by luminescent nanoparticles, and the “drug” function—by a biologically active substance deposited on the surface of nanoparticles.

The idea behind the presented investigation is to synthesize and study the functional properties of hybrids combining photoactive bioactivity and localizable luminescence. It should be noted that, so far, there are no reports in the literature of similar multifunctional hybrid nanomaterials demonstrating the combination of all three of the following properties (i) bioactivity, (ii) photoactivation, and (iii) luminescence. Currently, groups of substances possessing only a combination of “bioactivity–photoswitching” or “photoswitching–luminescence” properties have been reported [16,17,18].

As a simultaneously photoactive and bioactive substance, we chose a phosphorylated 2-arylaminomalonate compound with a Cl substituent in the phenyl ring (hereafter denoted as PhAM) [10]. The choice was determined by the large difference in butyrylcholinesterase (BChE) inhibition for the conformational states of the transition that can be stimulated by laser irradiation [10].

To perform the luminescence-related functions of “spatial localization” in the created hybrids, carbon quantum dots (CQDs) were chosen. CQDs are characterized by a combination of unique properties and have prospects for wide-ranging applications in pharmacology and biomedicine [19]. CQDs are small (2–10 nm) and highly soluble in water; they show photostable intense photoluminescence and are biocompatible, nontoxic, and can overcome natural biological barriers [19]. The CQD’s surface can be easily functionalized to adapt to specific problems [20]. It should be noted that the vast majority of the CQD-based hybrids created so far usually perform only one or both of two functions: (i) photoluminescence enabling imaging, and (ii) transport of drugs/genes/antiviral compounds [21,22,23,24,25,26,27,28,29,30,31,32]. We did not find any publication in which hybrids composed of CQDs and other compounds were used for photoactivation, which is an essential advance for photopharmacology.

In this study, a new CQDs@phosphonate hybrid nanomaterial is presented. We first created a hybrid that simultaneously performs four important functions: (1) ‘”bioactivity”; (2) photoactivation; (3) luminescence spatial localization; and (4) bioactivity state detection. The manuscript describes the process of synthesis, characterization, and testing of the new material. Obviously, such a nearly all-in-one material tested successfully in the lab, as reported here, might well initiate a major breakthrough in photopharmacology research.

## 2. Materials and Methods

### 2.1. Materials

Citric acid (Sigma Aldrich, Singapore, CAS Number: 77-92-9) and ethylenediamine (Acros Organics, Geel, Belgium, CAS number: 107-15-3) were used for CQD synthesis.

Diethyl 2-(dimethoxyphosphorylethynyl)-2-(4-clorinephenylamino) malonate was prepared by the reaction of chloroethynylphosphonate with aromatic secondary diethyl 2-[(4-clorinephenyl)amino]malonate. Diethyl 2-[(4-chlorinephenyl)amino]malonate was prepared from diethyl 2-bromomalonate and the 4-chloroaniline via the procedure adopted from [33]. Chloroacetylenephosphonic acid diethyl ester (*Choroethynylphosphonate*) was synthesized in our laboratory from dichloroacetylene etherate obtained by the method reported in [34] and triethyl phosphite. Diethyl bromomalonate (92%, Sigma-Aldrich, St. Louis, MI, USA), 4-Chloroaniline (99.8%, Sigma-Aldrich), acetonitrile (anhydrous, 99.8%, Sigma-Aldrich), Silica gel 60 (0.04–0.063 mm for column chromatography (230–400 mesh ASTM) Millipore, Burlington, MA, USA), n-Hexane (Laboratory Reagent, ≥95%, Sigma-Aldrich), ethyl acetate (Pure, Lenreaktiv, St. Petersburg, Russia) were used for PhAM synthesis.

Butyrylcholinesterase from horse blood plasma (EC 3.1.1.8), activity 264 U mg^−1^ (Sigma-Aldrich), bovine serum albumin (BSA) (Sigma-Aldrich), butyrylthiocholine chloride (Sigma-Aldrich); HEPES buffer solution (HEPES 0.005 M + KCl 0.003 M, pH 7.5) (Sigma-Aldrich); KMnO_4_, Mn(Ac)_2_·4H_2_O, K_2_CO_3_ (anhydrous, reagent grade, ≥98%, powder, −325 mesh, Sigma-Aldrich) were used for measurements of biological activity.

### 2.2. Methods

#### 2.2.1. Transmission Electron Microscopy

The structure and composition of CQDs were studied by transmission electron microscopy (TEM) (FEI Tecnai Osiris, Hillsboro, OH, USA) using the annular dark-field imaging method of sample mapping of scanning electron microscopy (HAADF STEM) (FEI Tecnai Osiris, Hillsboro, OH, USA) on a Tecnai Osiris microscope (Thermo Fisher Scientific, Waltham, MA, USA) at an accelerating voltage of 200 kV.

#### 2.2.2. X-ray Photoelectron Spectroscopy

The element composition of CQDs was studied using X-ray photoelectron spectroscopy (XPS) with AxisUltraDLD (Kratos, Manchester, UK) and LAS-3000 (Riber, Bezons, France) spectrometers. Photoelectrons were excited by X-ray radiation from an aluminum anode (AlKα = 1486.6 eV) at a tube voltage of 12 kV and an emission current of 20 mA. The photoelectron peaks were calibrated using the C 1 s carbon and N 1 s nitrogen lines with binding energies of 285 and 400 eV, respectively.

#### 2.2.3. Dynamic Light Scattering (DLS)

Measurements of the sizes/zeta potential of CQDs in suspensions were performed using the Malvern Zetasizer NanoZS instrument (Malvern, Worcestershire, UK).

#### 2.2.4. NMR

The structure of the product was confirmed by the data of ^1^H, ^13^C, ^31^P spectroscopy recorded with a Bruker Advance 400 spectrometer (Bruker, Mannheim, Germany) [400.13, 100.61, 40.54 MHz], the residual signals of the solvent (CDCl_3_) were used as an internal reference.

#### 2.2.5. Absorption Spectroscopy

The optical density spectra were recorded using a Shimadzu UV-3600 spectrophotometer (Shimadzu, Kyoto, Japan) in the wavelength range of 200–500 nm with a resolution of 1 nm (scanning speed—medium).

#### 2.2.6. Photoluminescence Spectroscopy

The photoluminescence spectra of the samples were obtained using a Shimadzu RF-6000 fluorimeter (Shimadzu, Kyoto, Japan). Additional processing of the spectra consisted in smoothing over 10 points using a moving average. The steady-state photoluminescence spectra were measured using a modular spectrofluorometer Fluorolog-3 (Horiba, Osaka, Japan) with a continuous wave Xe lamp as an excitation source. Luminescence kinetics measurements were carried out using LED340 and LED390 (pulse width 1.1 ns, repetition rate 1 MHz). Lifetimes were calculated using the TCSPC technique. All measurements were performed with samples with a CQD concentration of 6.6 × 10^−2^ mg/mL at room temperature. Excitation intensities used for steady-state and kinetic photoluminescence measurements were very low and did not affect the samples.

#### 2.2.7. FTIR Absorption Spectroscopy

The FTIR spectrum of CQD was recorded using a Bruker Invenio R FTIR spectrometer (Bruker, Mannheim, Germany) using an attenuated total reflection (ATR) accessory with a diamond crystal. The resulting spectrum was obtained by averaging 20 scans with a spectral resolution of 4 cm^–1^.

The FTIR spectra of hybrids were obtained at Nicolet8700 (Thermo Fisher Scientific; Waltham, MA, USA) using an ATR accessory with a diamond crystal. To obtain the FTIR spectra, the spectrometer configuration with a XT-KBr beam splitter and liquid nitrogen cooled mercury cadmium telluride (MCT-A) detector was used. For the solutions under investigation, the spectra were obtained in the region 650–4000 cm^−1^ with a resolution of 4 cm^−1^. In the obtained spectra, the Blackman–Harris apodization function was used and the Mertz phase correction was applied. Due to the low concentration of the investigated substances, the subtraction of the contribution of CQD in isopropanol (see Figure S1) was performed using Origin 9 (OriginLab Co., Northampton, MA, USA) software. Such mathematical procedures reveal any possible interaction between components of hybrids.

#### 2.2.8. BChE Activity Measurements

**Scheme 1:**
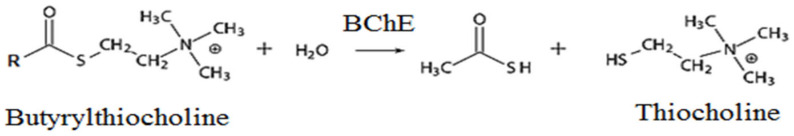
Enzymatic hydrolysis of butyrylthiocholine.

The activity of butyrylcholinesterase (BChE) is the initial rate of biocatalytic hydrolysis of butyrylthiocholine, which is determined by the accumulation of thiocholine using a thiol-sensitive sensor (“BVT”, Strážek, Czech Republic). The response to thiocholine formed during the enzymatic hydrolysis of butyrylthiocholine was recorded in the product accumulation mode (Scheme 1).

The BChE activity before and after inhibition was measured using an IPC-micro neurotoxin amperometric analyzer with integrated MnO_2_-modified planar electrodes [35,36].

To determine the inhibiting ability of the test substance, the stock solution of the BChE (1 mg/mL), and the substrate butyrylthiocholine chloride (0.5 M) were initially prepared. Then, an aliquot of the inhibitor (PhAM) (selected so that the concentration in the cell was 10^–3^:10^–7^ M), dissolved in the HEPES buffer containing BSA (1 mg/mL), pH 7.5, was placed in a microtube with a buffer solution. Incubation with the substrate was carried out for 10 min (in this interval, the enzymatic hydrolysis reaction is linear). The control (blanc) analysis—the preliminary measurements of BChE activity without inhibitor—was performed to normalize the enzyme activity before each inhibition measurement. In accordance with the literature data, the measurement inaccuracy of an IPC-micro neurotoxin analysis does not exceed 10% [35,36].

#### 2.2.9. Laser Irradiation of the Samples

Laser irradiation of the samples was performed with a Coherent MBD 266 solid-state laser (Santa Clara, CA, USA) (continuous wave, λ = 266 nm, power 120 mW) with a defocused laser beam (d = 10 mm) in a 1 cm thick quartz cell. The laser irradiation time was 30 min in all these experiments.

#### 2.2.10. Testing of CQD@PhAM Hybrids Functionality on Biological Models

The experiments with biological objects were carried out on a specially developed experimental setup. A diode laser was used as a source of excitation of CQD to initiate photoluminescence of the hybrids (λ = 405 nm, the power on the biological sample was 5 mW). The laser radiation was focused by a lens onto the surface of a sample placed on a glass slide. The reflected signal (elastic scattering + luminescence) was collected at the entrance slit of a monochromator (Acton 2500i, focal length 500 mM, grating 900 g/mm, the width of the entrance slit 250 µm). An Omega 420ALP edge filter (SKU: W3726) was used to reduce the contribution of scattered light. The obtained photoluminescence spectra were recorded using a PMT (Hamamatsu, H-8259-01). As a model experiment with a complex biological sample, a cut-out piece of chicken breast (across muscle fibers) was used. CQD and Hybrid4 CQD@PhAM were injected with a syringe into the chicken breast to a depth of ca. 1 mm. Part of the substance was poured onto the surface of the chicken breast; then the surface was carefully blotted with fat-free napkins.

## 3. Results

### 3.1. Phosphonates Synthesis

**Figure 1:**
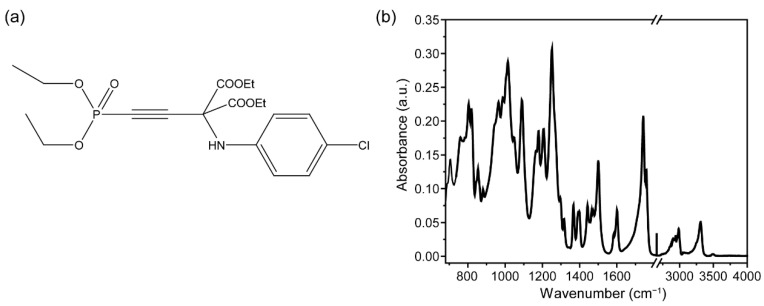
(**a**) Chemical structure of phosphonate—phosphorylated arylaminomalonate with Cl, (**b**) FTIR spectrum of phosphonate.

The phosphorylated arylaminomalonate compound (Figure 1a) with a Cl substituent in the phenyl ring was prepared according to a protocol reported earlier [37]. The chloroethynylphosphonates reacted with aromatic secondary diethyl 2-[(4-clorinephenyl)amino]malonate under mild conditions. The final product, diethyl 2-(dimethoxyphosphorylethynyl)-2-(4-clorinephenylamino)malonate, is a crystalline substance, readily soluble in the many organic solvents. The ^31^P NMR chemical shift of the phosphonate was observed in a strong field at −8.52 ppm. The ^31^P, ^1^H, and ^13^C NMR spectra and their description for the PhAM compound are presented in Figures S2–S4.

The FTIR spectrum of a synthesized sample includes the following peaks which confirm the synthesis of the target compound: 705, 804, 855, 1014, 1090, 1180, 1207, 1252, 1500, 1601, 1741,1759, 2987, and 3309 cm^−1^ (Figure 1b).

### 3.2. Carbon Quantum Dots Synthesis and Characterization

CQDs were synthesized by the hydrothermal method. Aqueous solutions of citric acid (3.36 g of citric acid power diluted in 19 mL of deionized water) and ethylenediamine (1.08 mL) were used as precursors (precursors ratio 1:1). The prepared mixture of precursors was placed in a Teflon container, which, in turn, was placed in a steel autoclave. Next, the sample was kept in a muffle furnace for 2 h at a temperature of 190 °C. To remove the larger fraction, the resulting highly concentrated aqueous suspension of CQDs was passed through a membrane filter with a pore diameter of 0.22 μm.

**Figure 2:**
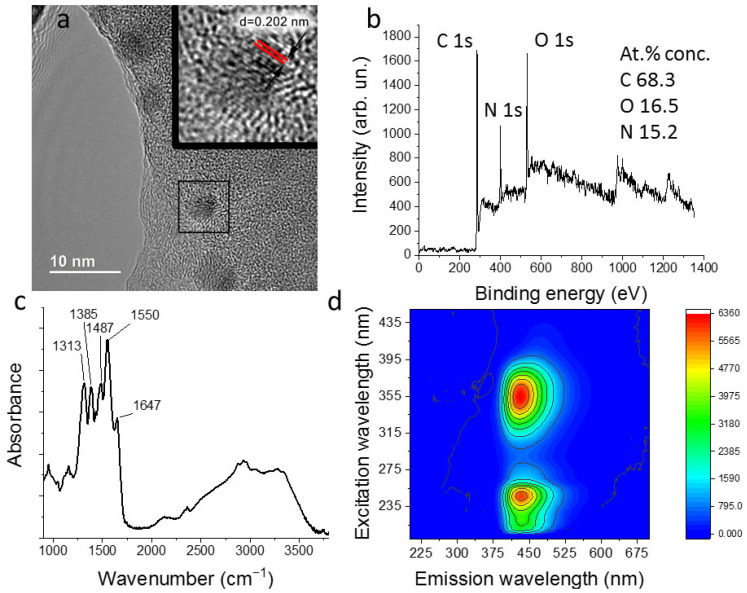
(**a**) HRTEM image of the synthesized CQDs, (**b**) XPS spectrum of the CQDs powder, (**c**) FTIR absorption spectrum of the synthesized CQDs. (**d**) Excitation/emission photoluminescence matrix of the CQDs suspension in isopropanol. The concentration of CQDs is 5 × 10^−3^ mg/mL.

According to the TEM results, the synthesized CQDs are carbon nanoparticles with an average size of 7.0 ± 0.5 nm (Figure 2a). One can see the lattice fringes with the lattice parameter is around 0.202 nm, corresponding to the (102) lattice fringes of graphite. For further studies, the CQDs were first transferred from an aqueous suspension to a dry form by lyophilization of the sample and then suspended in isopropyl alcohol. In isopropanol, the hydrodynamic particle size obtained by DLS was 79.4 ± 11.6 nm (Figure S5) with a zeta potential value of −7.8 ± 2.6 mV. A comparison of the values of the hydrodynamic particle size and the CQDs sizes obtained by HRTEM microscopy allows us to conclude that CQDs in isopropanol tend to aggregate. During the research, the formation of bigger aggregates of CQDs was not detected.

According to the XPS studies (Figure 2b and Figure S6), synthesized CQDs consist of carbon atoms (68.3%), oxygen atoms (16.5%), and nitrogen atoms (15.2%). The results of XPS and FTIR absorption spectroscopy (Figure 2b,c and Figure S6) indicate that the CQDs surface is polyfunctional. In the fingerprint region of the FTIR absorption spectrum, there are bands with maxima at 1313 cm^−1^ (corresponding to stretching ν(C-O), bending δ(C-H) vibrations), 1385 cm^−1^ (corresponding to δ(C-H), ν(C-O) vibrations), 1487 cm^−1^ (corresponding to ν(N=O), ν(O-O), δ(N-H) vibrations), 1550 cm^−1^ (corresponding to δ(N-H), ν(C-N), ν(C=C) vibrations) and 1647 cm^−1^ (corresponding to ν(C-N), ν(C=O), and δ(O-H) vibrations). In the high-frequency region of the spectrum (2700–3800 cm^−1^), bands attributed to the stretching vibrations of ν(C-H), ν(N-H), and ν(O-H) are observed [38]. The photoluminescence (PL) excitation/emission matrix of CQDs is shown in Figure 2b. As can be seen from the presented data, two separate maxima are observed in the PL excitation/emission matrix at wavelengths of (245/433) nm and (355/431) nm.

### 3.3. Phosphonate and Carbon Quantum Dots Hybrids

Phosphonate and carbon quantum dots hybrids (CQD@PhAM) were prepared by steering the components at ambient conditions for different times. To study the effect of the conjunction of the components on the final photo pharmacological properties, we prepared several samples based on phosphonate (PhAM) and carbon quantum dots (CQD)—(1) Mix CQD-PhAM, (2) Hybrid1 CQD@PhAM and (3) Hybrid4 CQD@Ph. Mix CQD-PhAM was prepared as a result of adding PhAM powder to the CQD suspension in isopropanol without stirring: the concentration of CQD suspension in isopropanol was 1 mg/mL and the concentration of phosphonate was 10^−2^ M. Hybrid1 CQD-PhAM was obtained by stirring the sample Mix with a magnetic stirrer for 1 h. Hybrid4 CQD-PhAM was obtained as a result of 4 h of stirring. Then the obtained samples were studied with absorption, luminescence, and FTIR spectroscopies as well as IPC-micro neurotoxin amperometric analysis.

**Figure 3:**
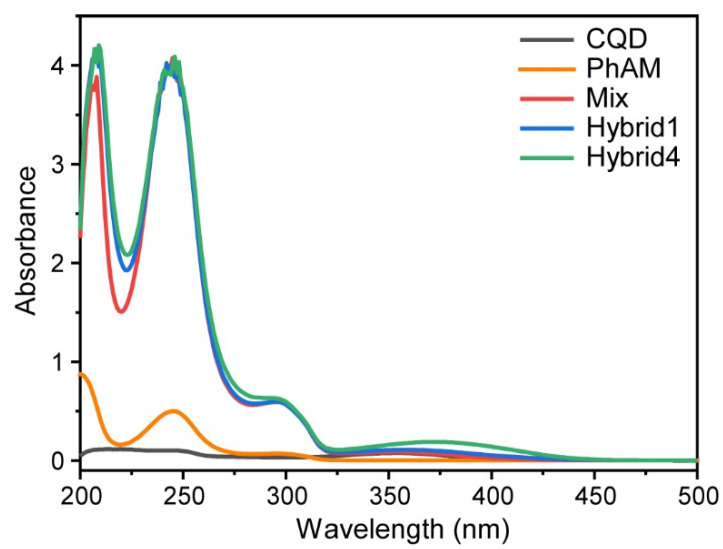
Absorption spectra of reference samples CQD (black), PhAM (orange), and the prepared samples Mix (red), Hybrid1 (blue), and Hybrid4 (green). The concentration of CQDs was 3.3 × 10^−2^ mg/mL, concentrations of phosphonates in Mix and Hybrid samples were 3.3 × 10^−4^ M. PhAM sample concentration was 3 × 10^−5^ M.

Figure 3 shows the absorption spectra of the prepared samples (Mix, Hybrid1, Hybrid4) and reference samples of CQD and PhAM. CQD display peaks with maxima in the region of 240 nm (transitions π–π* of C=C band [39]) and 353 nm (n-π* transition of multi-conjugate C=O of the CQDs [40]). Also, a peak at 210 nm was observed that is most likely an artefact, arising from the UV cut-off of solvent at 205 nm [41].

One can see that the spectra of Mix, Hybrid1, and Hybrid4 are characterized by absorption bands at 240 and 290 nm, typical for phosphorylated 2-arylaminomalonate compound (orange line in Figure 3). The absorption band in the region 350–400 nm is associated with CQD. It is interesting to note regular spectra modification for the samples Mix, Hybrid1, and Hybrid4—a gradual increase in the gap between the bands 210 nm, 240nm, and 290 nm; and a shift of CQD related band from 353 to 380 nm. The observed peculiarities testify to an increase in the interaction between the components PhAM and CQD for the mixed and hybrid samples and confirm hybrid formation.

**Figure 4:**
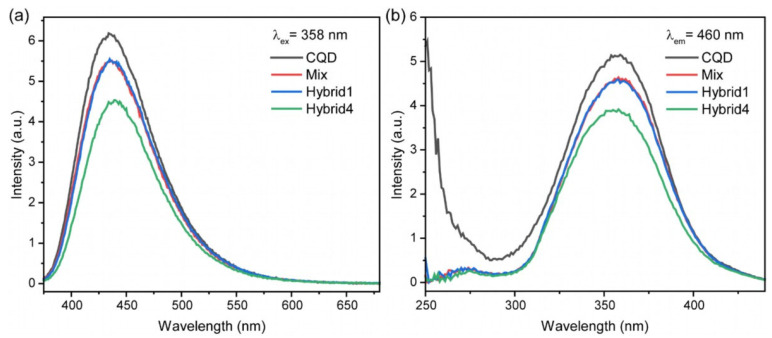
(**a**) Emission (λ_ex_ = 358 nm) and (**b**) excitation spectra (λ_em_ = 460 nm) of CQD (black), Mix (red), Hybrid1 (blue), and Hybrid4 (green). The concentration of CQDs in all samples was 6.6 × 10^−2^ mg/mL.

Keeping in mind the pronounced luminescence properties of CQD, we studied the effect of the conjunction of the components on the luminescence behavior. Emission and excitation spectra for the samples CQD, Mix, Hybrid1, and Hybrid4 are presented in Figure 4a,b, respectively. The introduction of phosphonates into the system leads to a luminescence intensity decrease. Luminescence spectra of Mix and Hybrid 1 look similar, while Hybrid4 shows a distinct decrease. Along with a decrease in the intensity of the emission and excitation bands, there is a slight shift of the bands towards the red spectral region (for emission) and blue region (for excitation). Therefore, the luminescence spectroscopy supports UV-VIS absorption data towards the formation of hybrids.

**Figure 5:**
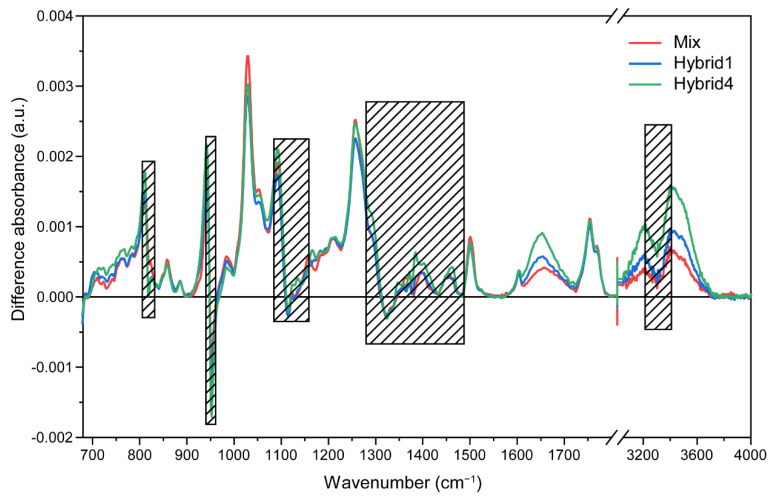
Difference IR absorption spectrum with subtraction of the contribution from CQD in isopropanol for Mix (red), Hybrid1 (blue), and Hybrid4 (green). The shaded regions correspond to solvent absorption.

FTIR absorbance spectroscopy is a sensitive technique allowing one to observe structural changes, which can take place during hybrid formation. To provide the observed changes in the PhAM component more clearly, the FTIR spectrum of CQD in isopropanol was recorded (Figure S1) so that the contribution of CQD can be subtracted. When comparing the resulting difference IR absorption spectra of the Mix with the spectra of the Hybrid1 and Hybrid4 (Figure 5), a monotonous change in the absorption in the region v(C=O) and δ(H_2_O) (1620–1700 cm^−1^), as well as in the region of stretching hydrogen vibrations in O-H and N-H bonds (3100–3600 cm^−1^) was observed [42]. In addition, Hybrids display a lower absorption for the band with a maximum at about 1024 cm^−1^ (significant contribution from stretching vibrations in the P-O and C-O bonds in PO_3_ and COO groups as well as stretching vibrations of C-O bonds in P-O-C and C-O-C parts) [9,10,15,42]. Thus, the main changes occur in the IR absorption spectra of phosphonates’ and C=O bond-containing polar groups, which are primary candidates for involvement in the process of PhAM adsorption on CQDs. The obtained results confirm the active interaction of PhAM with CQDs, the degree of which increases with the increase in the time of stirring during the synthesis of hybrids.

**Figure 6:**
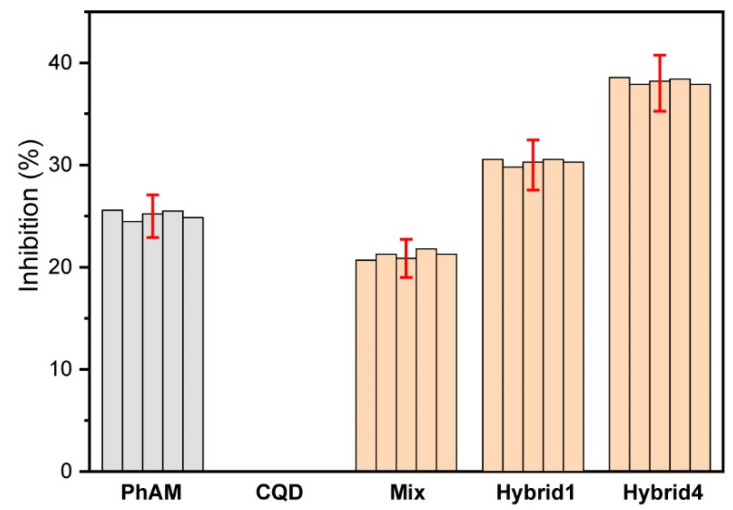
BChE inhibition value for PhAM, CQD, Mix, Hybrid1, and Hybrid4. Data repeatability was checked for five samples.

In such a way, absorption, luminescence, and IR spectroscopy reveal the conjunction of the components CQD and phosphorylated arylaminomalonate, and the formation of a hybrid. Then biological activity towards BChE inhibition was measured for Mix, Hybrid 1, and Hybrid 4, and for reference samples—PhAM and CQD. It was found that the CQD isopropanol solution does not have any biological activity towards BChE; PhAM showed BChE inhibition of 25%. The mix sample shows no effect of the CQD on the biological activity of PhAM within measurement error (Figure 6). Hybrid1 has increased inhibition in comparison with Mix—30% and 21%, respectively. Hybrid4 demonstrates even higher growth–up to 38%. Thus, the large difference in BChE inhibition values for PhAM, Mix, Hybrid1, and Hybrid4 indicates a change in the spatial structure of the PhAM in the hybrids and, as a result, a change in the geometry of the structural interaction of the phosphonate molecule with the active center of the BChE enzyme.

Summarizing the results of PhAM, CQD, Mix, Hybrid1, and Hybrid4 samples investigation with comprehensive techniques, one can conclude that the process of the conjunction of PhAM and CQD by means of prolonged stirring results in the formation of hybrids possessing chemical interaction of the components via polar groups of PhAM. The properties of the resulting hybrids differ from that of the individual components. This is manifested by changes in the FTIR spectra, by a decrease in the luminescence intensity, and by an increase of BChE inhibition for Hybrid1 and Hybrid4 in comparison with PhAM, CQD, and Mix samples. In such a way, we demonstrated for the first time the formation of hybrids from bioactive organophosphorus compounds and luminescent CQD.

However, the chosen organophosphorus compound is characterized by its ability to change the conformation after illumination with resonant laser irradiation [10]. Our earlier investigations of phosphorylated arylaminomalonate demonstrated a photoinduced transition between conformational states of PhAM, resulting in an increase of BChE inhibition from 24% to 90%. Keeping in mind that the properties, luminescence, and BChE inhibition of CQD@PhAM hybrid are sustained, the investigation of their change after laser irradiation is a curious question to be addressed as the next item.

### 3.4. Laser Irradiation and Characterization of CQD@PhAM Hybrids

**Figure 7:**
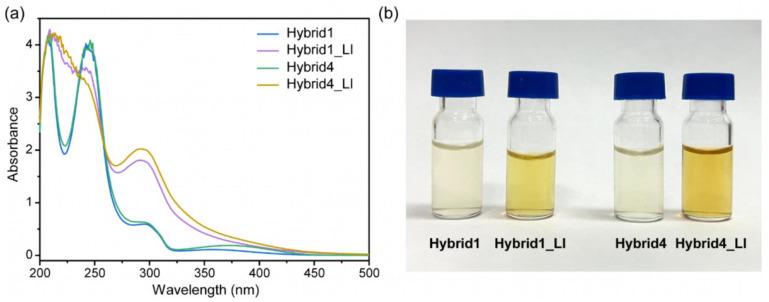
(**a**) Absorption spectra of Hybrid1 and Hybrid4 before and after laser irradiation with laser irradiation at λ_ex_ = 266 nm during 30 min, (**b**) photo of isopropanol solution of Hybrid 1 and Hybrid 4 before and after laser irradiation (Hybrid1_LI, Hybrid4_LI). The concentrations of phosphonates in all samples were 3.3 × 10^−4^ M.

In accordance with the absorption spectra presented in Figure 3, PhAM and Hybrids CQD@PhAM are characterized by several bands; however, the highest absorption is observed at 250 nm. That is why solid-state laser Coherent MBD 266 (Coherent) (λ_ex_ = 266 nm, power 120 mW) was chosen for irradiation of CQD@PhAM hybrids. Figure 7a shows absorption spectra of Hybrid1 and Hybrid4 before and after 30 min laser irradiation (Hybrid1_LI, Hybrid4_LI). In both cases, one can see some pronounced changes but also some similarities in the absorbance spectra. Laser irradiation results in a decrease of the 250 nm peak and a simultaneous increase of the absorption band at 300 nm. Another interesting feature is the growth of the absorption shoulder in the range of 300–450 nm, thus, resulting in the hiding of the CQD-related band at 350–370 nm. These changes resulted in a bright brown coloring of the hybrids in solution (Figure 7b).

**Figure 8:**
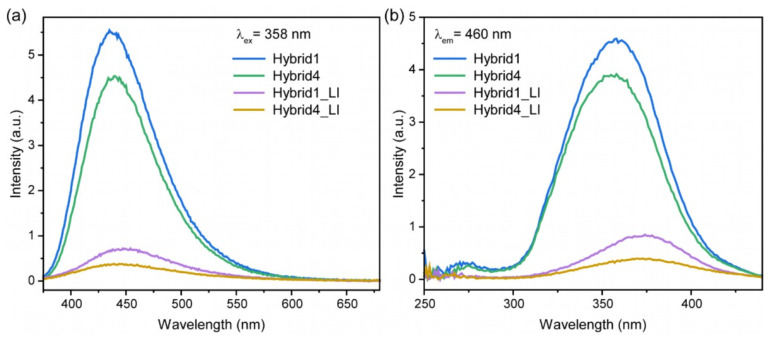
(**a**) Emission (λ_ex_ = 358 nm) and (**b**) excitation (λ_em_ = 460 nm) spectra of Hybrid1 and Hybrid4 before and after laser irradiation (Hybrid1_LI, Hybrid4_LI). The concentration of CQDs in all samples was 6.6 × 10^−2^ mg/mL.

The effect of laser irradiation on the photoluminescence properties of Hybrids CQD@PhAM is shown in Figure 8. One can see a sharp drop in emission intensity for both Hybrid1 and Hybrid4 after laser irradiation (Hybrid1_LI, Hybrid4_LI); see Figure 8a. Noteworthy, the observed intensity decrease was higher for Hybrid4 as compared to Hybrid1 (12 vs. 8 times). Moreover, a slight red shift of the emission band was observed. The excitation spectra display a similar behavior, including a slight red shift of the excitation band (Figure 8b). The observed changes in the photoluminescence can be explained by the inner filter effect: the increased absorption of the hybrids after irradiation (Figure 7) causes the decrease of both excitation and emission radiation intensities, the latter of which is more pronounced for the smaller wavelength. This, in turn, causes the observed spectral shifts.

**Figure 9:**
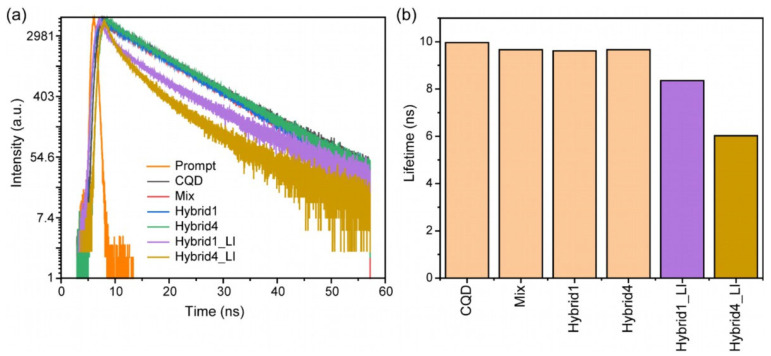
(**a**) Luminescence decay curves and (**b**) observed lifetimes of CQD, Mix, Hybrid1, and Hybrid4 before and after laser irradiation.

Important information about hybrid formation and the effect of laser irradiation can be extracted from luminescence kinetics measurements. Figure 9 presents kinetic decay curves and observed lifetimes for the studied samples CQD, Mix, and Hybrids before and after laser irradiation. Decay curves of CQD, Mix and non-irradiated Hybrids show mono-exponential behavior. It is interesting to note that hybrid formation does not change the lifetime (~9.7 ns), while laser-irradiated Hybrids demonstrate significant changes in the shape of the decay curves. In the latter case, a biexponential model was required in the fitting to match the experimental data. An average lifetime τ_av_, was calculated for a fair comparison with samples before irradiation: $\tau_{av}=\frac{I_{1}\tau_{1}^{2}+I_{2}\tau_{2}^{2}}{I_{1}\tau_{1}+I_{2}\tau_{2}}$, where *I*_1_ and *I*_2_ are the amplitudes of the exponential terms with decay constants (lifetimes) *τ*_1_ and *τ*_2_ [43]. The analysis of the lifetimes resulting from fitting the data reveals a dramatic decrease in lifetime after laser irradiation (Table S1). This fact can be explained by non-radiative energy transfer from CQD to PhAM inside one Hybrid, or by radiative energy transfer via absorbance of emitted luminescence by neighboring Hybrid. The observed strong difference in luminescence lifetimes for Hybrids before and after laser irradiation can be used as an efficient indicator of hybrid state—“non-illuminated” or “illuminated”.

**Figure 10:**
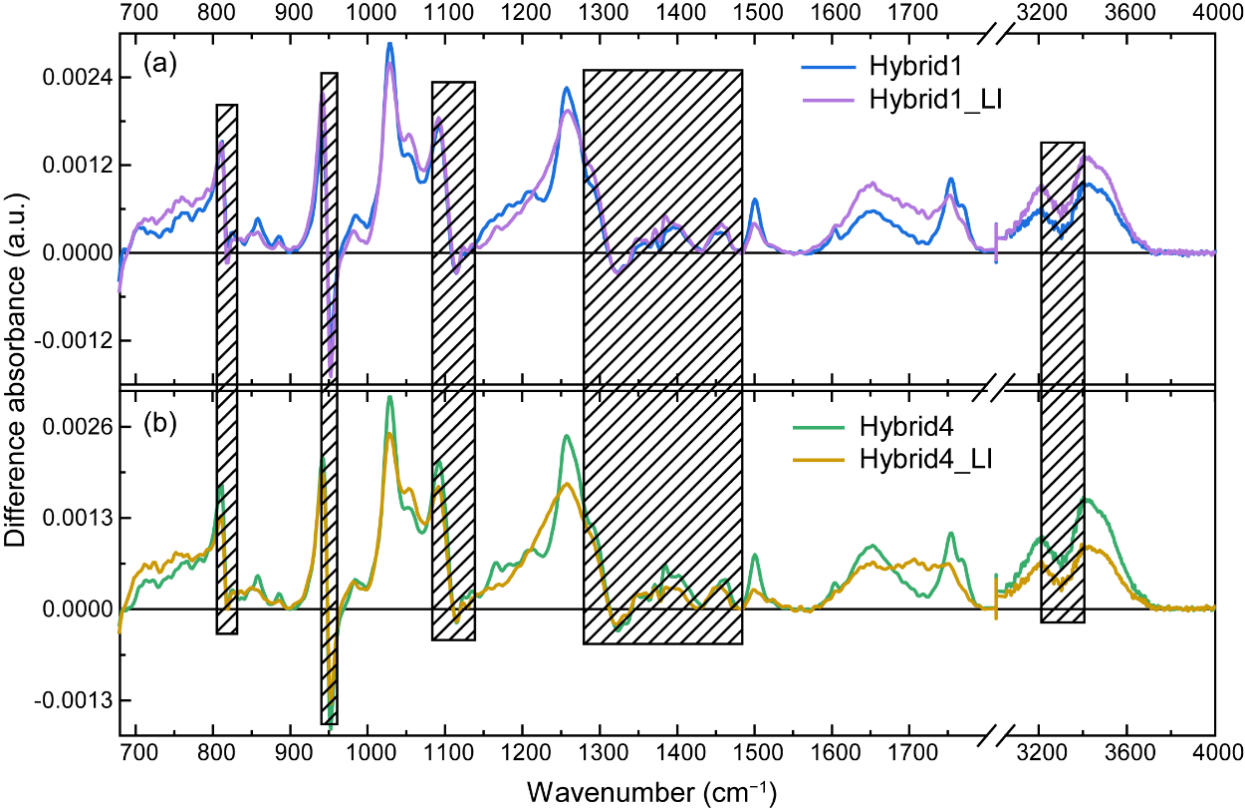
(**a**) Difference IR absorption spectrum of Hybrid1 before (blue) and after Hybrid1_LI (violet) laser irradiation; (**b**) difference IR absorption spectrum of Hybrid4 before (green) and after Hybrid4_LI (orange) laser irradiation. The shaded regions correspond to solvent absorption.

To study structural changes in the CQD@PhAM hybrids after laser irradiation, which significantly affects absorption and luminescence properties, FTIR spectroscopy was applied. Similar to FTIR spectroscopy provided earlier, the spectrum of CQD in isopropanol was subtracted from the spectra under study. When comparing the spectra of Hybrid1 and Hybrid4 before and after irradiation (Figure 10), a number of simultaneous changes in the peak intensities can be noted. Their interpretation is based on the studies reported elsewhere [9,10,15,42,44]. After laser irradiation, there is a lower absorption for the following peaks: 1750 and 1768 cm^−1^ (v(C=O) in COO groups), 1500 cm^−1^ (δ(NCH) and δ(CCH) in the benzene ring), 1288 cm^−1^ (v(P=O) in the PO_3_ group and v(C-O) inside the COO groups), 1028 and 860 cm^−1^ (v(C-O), δ(OCO) in and near COO groups and ρ(CH_3_)). At the same time, there is an increase in absorption for the peak at 1051 cm^−1^ (spectral region of v(C-O) in P-O-C). In addition, a new peak corresponding to v(C=O) appears at about 1705 cm^−1^, which differs from v(C=O) in PhAM and CQD. Noteworthy, C=O bond is highly polar and, therefore, sensitive to its environment and hydrogen bond formation. Hybrid4 displays more pronounced changes in comparison to Hybrid1. Thus, it can be assumed that during irradiation, changes occur as a result of PhAM-CQD interaction through the polar groups COO and PO_3_, and also through hydrogen bond formation.

**Figure 11:**
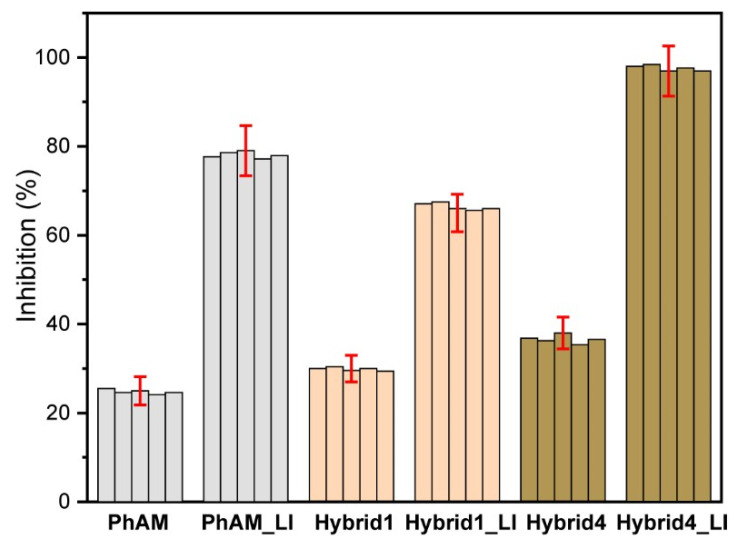
BChE inhibition value for PhAM, Hybrid1, and Hybrid4 before and after laser irradiation. Data repeatability was checked for 5 samples.

Checking the effect of laser irradiation on the bioactivity of hybrids, we have found that BChE inhibition is increased more than twofold and reached 65% (versus 29%) in the case of Hybrid1 and almost 100% (versus 38%) in the case of Hybrid4 (Figure 11). If we compare the biological activity of pure phosphonate before and after laser irradiation with the obtained hybrids, we can see that non-irradiated and irradiated Hybrid4 demonstrates higher activity than phosphorylated arylaminomalonate. It should be noted that CQD@PhAM Hybrid4 demonstrates not only the retention of biological activity and ability to photo-activate but also shows a large difference in BChE inhibition (more than 60%) before and after laser irradiation. Hybrid 1 shows a rather moderate difference with phosphonate which is probably a consequence of the hybrid structure not yet being complete after 1 h of stirring.

As subsequent experiments showed (Figure S7a), the samples Mix, Hybrid1, and Hybrid4, as well as laser irradiated Hybrid1 and Hybrid4, retain their bioactivity for a long time (90 days). For Hybrid4, there is only a slight decrease of BChE inhibition. By itself, phosphorylated arylaminomalonate also maintains stable values of biological activity. The BChE inhibition for non-irradiated and irradiated PhAM after 2 years is shown in Figure S7b.

### 3.5. Testing of CQD@PhAM Hybrids Functionality on Biological Models

The control of the location of the drugs in the organs and tissues of the body is a fundamental factor in the treatment of a wide list of localized diseases (skin diseases, ophthalmic problems) or diseases requiring site-specific drug delivery (Alzheimer, myocardial infarction, oncological diseases, etc.). The solution to this problem can be implemented using luminescent substances for drug mapping. However, the visualizing of luminescent objects in biological tissues can be difficult due to autofluorescence—the intrinsic property of cells. Thus, a study of the possibility of detecting a luminescent marker against the background of autofluorescence is an essential test for biomedical applicability.

**Figure 12:**
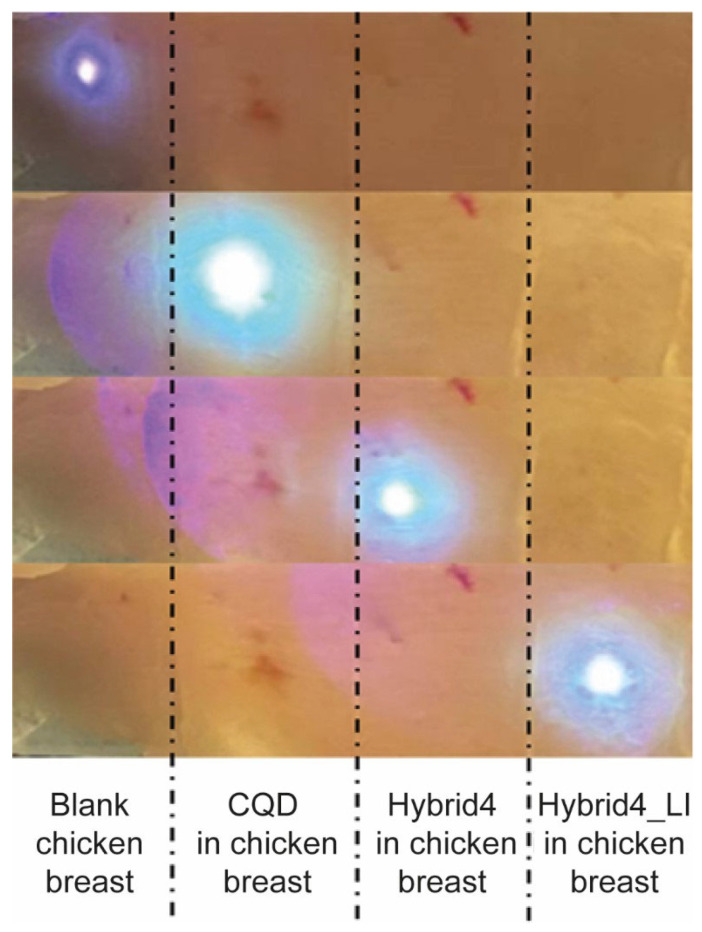
Images of laser irradiated chicken breast sample without injection and after CQD, Hybrid4, and Hybrid4_LI injection (from **left** to **right**).

As a biological object in our experiments, a cross-section cut out of a chicken breast (across muscle fibers) was used. CQD and Hybrid4 CQD@PhAM were injected with a syringe into the chicken breast to a depth of ca. 1 mm. Figure 12 demonstrates photos of laser irradiated (λ_ex_ = 405 nm) chicken breast sample without injection, and after CQD, Hybrid4, and Hybrid4_LI injection (from top to bottom). One can see clear visualization of CQD and Hybrid4 against the background of chicken breast autofluorescence (as seen without injection): CQD and Hybrid4 photoluminescence stands out not only in intensity but also in color. Hybrid4_LI photoluminescence also stands out against the background of biotissue autofluorescence; however, a strong decrease in the intensity as compared to CQD and Hybrid4 luminescence and the transformation of turquoise color to blue, similar to the color of autofluorescence, is clearly visible.

**Figure 13:**
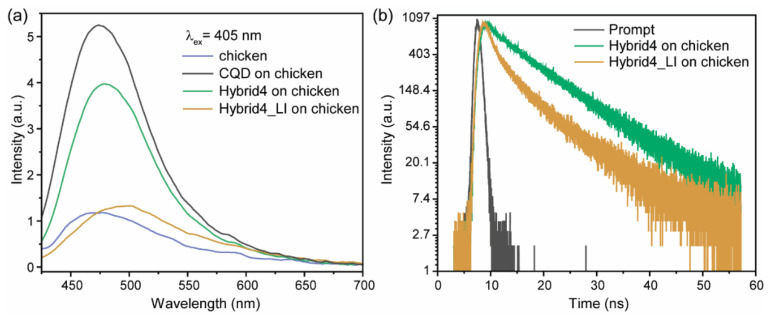
(**a**) Emission spectra of chicken breast, CQD, non-irradiated Hybrid4 and laser-irradiated Hybrid4_LI on the chicken breast, (**b**) kinetic decay curves of non-irradiated Hybrid4 and laser-irradiated Hybrid4_LI on the chicken breast.

The visual observation is confirmed by emission spectra of chicken breast, non-irradiated Hybrid4 and laser-irradiated Hybrid4_LI on the chicken breast (Figure 13a). Emission spectra were recorded upon 405 nm excitation. Chicken breast data demonstrate quite broad autofluorescence signal centered at about 470 nm. It should be noted that emission maxima of non-irradiated and laser-irradiated Hybrid4 on the chicken breast undergo significant spectral shift compared to isopropanol solution (Figure 8a), which can be explained by the change of excitation wavelength and environment [45,46]. Laser-irradiated Hybrid4_LI on the chicken breast displays a red shift of the emission band between 480 and 495 nm and a significant decrease in intensity as it was observed earlier for the isopropanol solution. The changes in the photoluminescence intensity and its color observed for Hybrids (see Figure 12) are in good agreement with the obtained spectra of the samples. Figure 13b presents kinetic decay curves of non-irradiated Hybrid4 and laser-irradiated Hybrid4_LI on the chicken breast measured at luminescence maxima 480 and 495 nm, respectively. In the former case, the experimental data can be fitted with a mono-exponential function, while the bi-exponential model is required in the case of Hybrid4_LI. Table S2 lists all fitted lifetime parameters.

It can be concluded that the change of the luminescence decay resulting from the fitting procedure can be used as a reliable indicator to determine the “non-illuminated” or “illuminated” hybrid state even in such a complex environment as biological tissue. Thus, for in vitro experiments, the observed features of non-irradiated and laser-irradiated CQD@PhAM hybrids (luminescence and kinetic decay) are preserved on the biological test sample. Moreover, in spite of unfavorable conditions—autoluminescence of the biosample in a close spectral region, the visualization of the localization of CQD@PhAM hybrids on the chicken breast is readily observed. Furthermore, the hybrid state “non-irradiated” or “irradiated” is readily distinguished by observation.

**Figure 14:**
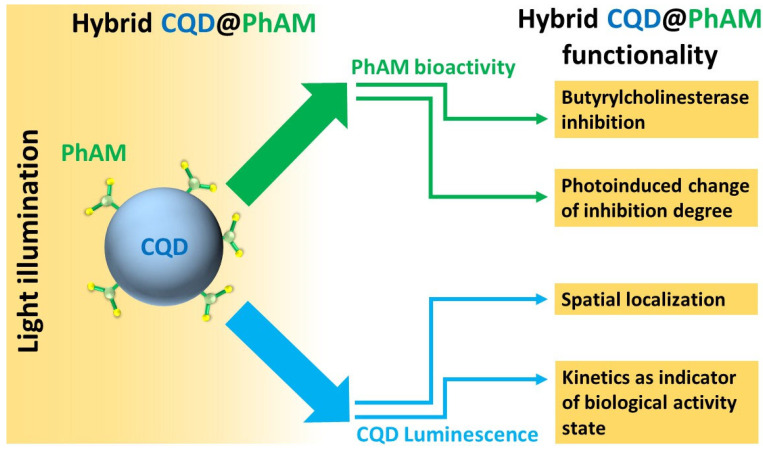
Scheme of CQD@PhAM Hybrids functionality.

In such a way, the series of experiments on laser irradiation of Hybrids results in considerable changes in critical properties—luminescence and bioactivity. Furthermore, the photoactivation ability typical for phosphonates is preserved for Hybrids, which allowed for obtaining compounds with highly different values of BChE inhibition before and after laser irradiation. An extremely important observation is the strong difference in luminescence kinetics behavior for non-irradiated and laser-irradiated Hybrids. It is revealed in a change of the decay curve shape and in significantly different lifetimes. It should be noted that the luminescence features are observed not only for in vitro experiments with Hybrids solutions but also for biosamples injected with Hybrids. In such a way, luminescence kinetics can be considered as an indicator of the state of biological activity of CQD@PhAM Hybrids—mono-exponential decay testifies to non-irradiated hybrid with moderate bioactivity, biexponential decay testifies to hybrid with “switched on” bioactivity. Thus, the obtained CQD@PhAM hybrids are unique multifunctional materials (Figure 14) combining several properties important for photopharmacology: (i) bioactivity towards butyrylcholinesterase inhibition, (ii) change of butyrylcholinesterase inhibition as a result of laser irradiation, (iii) localization by luminescence registered against the auto luminescence background of biological tissues, and (iv) luminescence kinetics as an indicator of state of biological activity of CQD@PhAM Hybrids.

## 4. Conclusions

In summary, we demonstrated for the first time the synthesis and characterization of organic-inorganic hybrids based on luminescent carbon quantum dots and a photoactive phosphorylated 2-arylaminomalonate compound. The synthesis of the hybrids was obtained in the course of stirring components at ambient conditions. The formation of hybrids was confirmed by complementary techniques—absorption, luminescence, FTIR spectroscopies, and bioactivity investigation—checking of butyrylcholinesterase inhibition. It was found that CQD@PhAM Hybrids are characterized by red-shifted bands and lower luminescence intensity in comparison to CQD, while BChE inhibition of Hybrids is higher than that for the phosphorylated 2-arylaminomalonate compound. FTIR data analysis revealed the formation of Hybrids by means of phosphonates adsorption on CQDs surface via polar groups. All the obtained results confirm the active interaction of PhAM with CQDs. The stability of the CQD@PhAM hybrids was confirmed by biological tests on butyrylcholinesterase inhibition after 90 days of storage at ambient conditions.

Further experiments were aimed at studying the Hybrid properties after exposure to laser irradiation with a wavelength of 266 nm (corresponding to the intrinsic absorption band of the phosphonate compound). It was interesting to discover that not only the luminescent properties and the bioactivity of the CQD@PhAM Hybrids were retained after laser irradiation but also the steep increase of BChE inhibition up to almost 100% and the simultaneous luminescence intensity decrease. In such a way, laser irradiation of Hybrids results in a considerable change of critical properties—luminescence and bioactivity. Furthermore, the photoactivation ability typical for phosphonates is preserved for Hybrids that allow obtaining compounds with highly different values of BChE inhibition in states before and after laser irradiation. A highly relevant observation is the strong difference in the luminescence kinetics behavior for non-irradiated and laser-irradiated Hybrids, which is revealed in a change in the shape of the decay curve and in significantly different lifetimes. In such a way, the luminescence kinetics can be considered as an indicator of the state of biological activity of the CQD@PhAM Hybrids—mono-exponential decay testifying to non-irradiated hybrid with moderate bioactivity, and biexponential decay testifying to hybrid with “switched on” bioactivity. These features were observed both for in vitro experiments and for a biological test sample—chicken breast. We have demonstrated that the CQD@PhAM hybrids luminescence can be registered against the autoluminescence background of biological tissues. Thus, the obtained CQD@PhAM hybrids are unique multifunctional materials providing all-in-one important functions for photopharmacology: (i) bioactivity toward BChE inhibition, (ii) ability to change the degree of inhibition as a result of laser irradiation, (iii) luminescence kinetics as an indicator of biological activity state and (iv) luminescence as a visualization of Hybrid spatial localization on the surface of a sample.

## Data Availability

The data presented in this study are available on request from the corresponding author.

## Supplementary Materials

The following supporting information can be downloaded at: https://www.mdpi.com/article/10.3390/nano13172409/s1, Figure S1: FTIR spectra of (a) isopropanol, (b) Mix with subtracted contribution of CQD in isopropanol, (c) PhAM in solid state; Figure S2: ^31^P NMR spectrum of the compound PhAM; Figure S3: ^1^H NMR spectrum of the compound PhAM; Figure S4: ^13^C NMR spectrum of the compound PhAM; Figure S5. CQDs size distribution in aqueous suspension obtained using the DLS method; Figure S6: High-resolution C1s (a), N1s (b), and O1s (c) XPS spectra of CQD; Figure S7: BChE inhibition value for (a) Mix, Hybrid1 and Hybrid4 and (b) PhAM before and after laser irradiation for a prolonged period; Table S1: Fitting parameters of decay kinetic curves for CQD, Mix, unirradiated and irradiated Hybrid1 and Hybrid4; Table S2: Fitting parameters of decay kinetic curves for unirradiated and irradiated Hybrid4 on the chicken breast.
