# Supplementary material for: All-in-One Photoactivated Inhibition of Butyrylcholinesterase Combined with Luminescence as an Activation and Localization Indicator: Carbon Quantum Dots@Phosphonate Hybrids
Source: Nanomaterials (Basel). 2023 Aug 25;13(17):2409. doi: 10.3390/nano13172409 (PMC10489800; doi:10.3390/nano13172409)
Supplement: Supplementary file 1 [file nanomaterials-13-02409-s001.zip › nanomaterials-2552388-supplementary.pdf]

nanomaterials

Supporting information

Article

All-in-One Photoactivated Inhibition of Butyrylcholinesterase Combined with Luminescence as an Activation and Localization Indicator: Carbon Quantum Dots@Phosphonate Hybrids

Gulia Bikbaeva [1,2], Anna Pilip [3], Anastasia Egorova [3,4], Ilya Kolesnikov [2], Dmitrii Pankin [2], Kirill Laptinskiy [5], Alexey Vervald [5], Tatiana Dolenko [5], Gerd Leuchs [6,*] and Alina Manshina [1,*]

1 Institute of Chemistry, St. Petersburg State University, St. Petersburg 199034, Russia; st086467@student.spbu.ru
2 Center for Optical and Laser Materials Research, St. Petersburg State University, St. Petersburg 199034, Russia; ie.kolesnikov@gmail.com (I.K.); dima-pankin@mail.ru (D.P.)
3 St. Petersburg Federal Research Center of the Russian Academy of Sciences (SPC RAS), Scientific Research Centre for Ecological Safety of the Russian Academy of Sciences, St. Petersburg 197110, Russia; anyta_273@mail.ru (A.P.); diekerze54@gmail.com (A.E.)
4 World-Class Laboratory, St. Petersburg State Technological Institute (Technical University), St. Petersburg 190013, Russia
5 D.V. Skobeltsyn Institute of Nuclear Physics, M.V. Lomonosov Moscow State University, Moscow 119991, Russia; laptinskiy@physics.msu.ru (K.L.); alexey.vervald@physics.msu.ru (A.V.); tdolenko@mail.ru (T.D.)
6 Max Planck Institute for the Science of Light, 91058 Erlangen, Germany
* Correspondence: gerd.leuchs@mpl.mpg.de (G.L.); a.manshina@spbu.ru (A.M.)

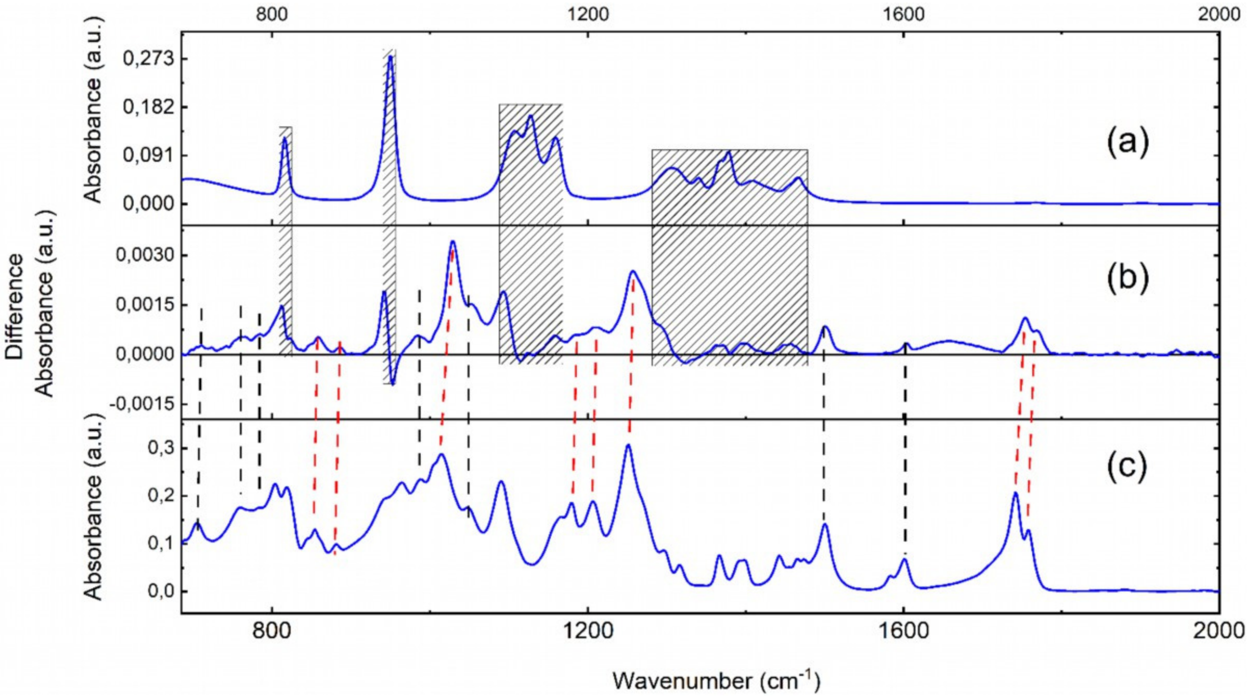

Figure S1. FTIR spectra of (a) isopropanol, (b) Mix with subtracted contribution of CQD in isopropanol, (c) PhAM in solid state.

NMR spectra of diethyl 2-(diethoxyphosphorylethynyl)-2-(4-chlorophenylamino) malonate:

^{1}H NMR spectrum, δ, ppm: 1.30 t (6H, CH_3, $^3J_{HH}$ 6.9 Hz), 1.32 t (6H, CH_3, $^3J_{HH}$ 7.5 Hz), 4.09 m (4H, CH_2OP, $^3J_{HH}$ 6.2 Hz, $^3J_{HP}$ 8.4Hz), 4.36 q (4H, OCH_2, $^3J_{HH}$ 7.1 Hz), 5.29 s (1H, NH), 6.72 d (2H, *o*-Ph-N,$^3J_{HH}$ 8.8 Hz), 7.17 d (2H, *m*-Ph-N, $^3J_{HH}$ 8.8 Hz). ^{13}C NMR spectrum, δC, ppm: 13.86 (CH_3), 15.96 d (CH_3,$^4J_{CP}$ 6,9 Hz), 63.47 d (P–C≡C–C, $^3J_{CP}$ 4,4 Hz,), 63.60 d (CH_2OP, $^2J_{CP}$ 5.5 Hz), 64.28 s (OCH_2), 76.94d (P–C≡C–C, $^1J_{CP}$ 287.2 Hz), 92.29 d (P–C≡C–C, $^2J_{CP}$ 48.6 Hz), 116.28 (*o*-Ph-N), 124.87 (*m*-Ph-N), 129.06 (*ipso*-Ph-N), 141.46 (*ipso*-Ph-Cl), 164.73 (C=O). ^{31}P NMR spectrum: δP -8.52 ppm.

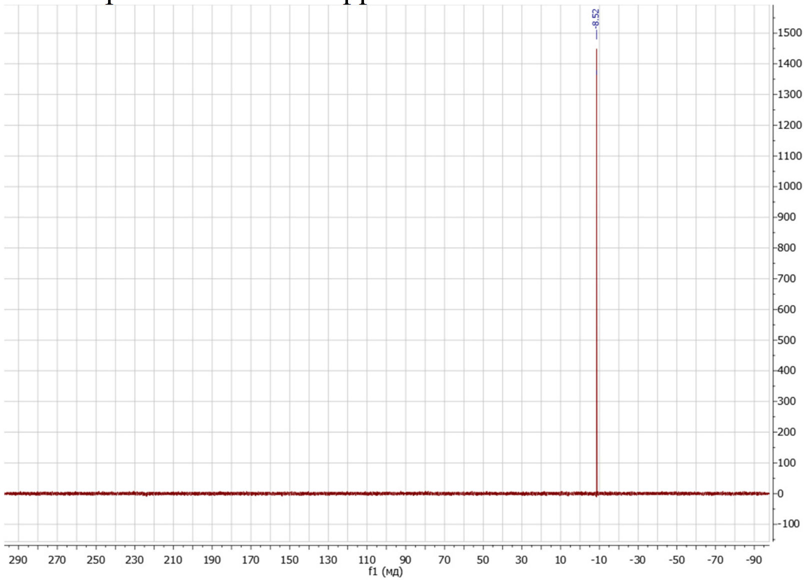

Figure S2. ^{31}P NMR spectrum of the compound PhAM

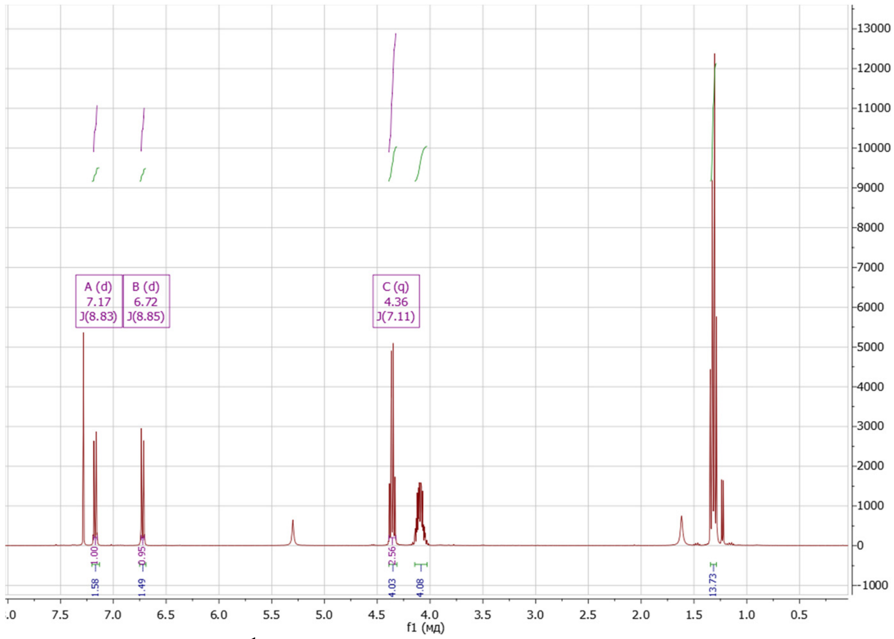

Figure S3. ^{1}H NMR spectrum of the compound PhAM

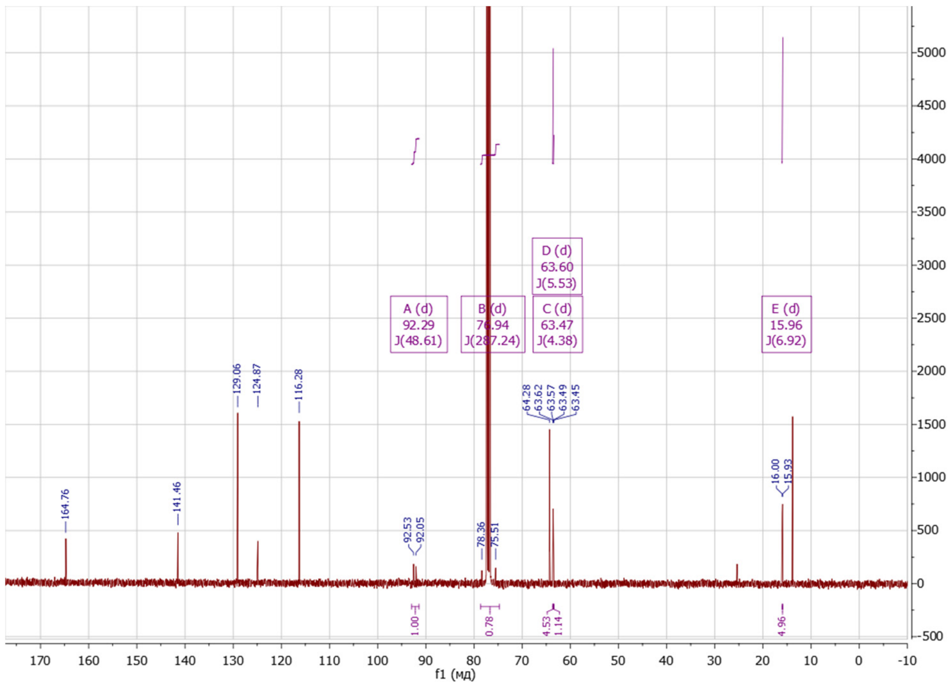

Figure S4. ^{13}C NMR spectrum of the compound PhAM

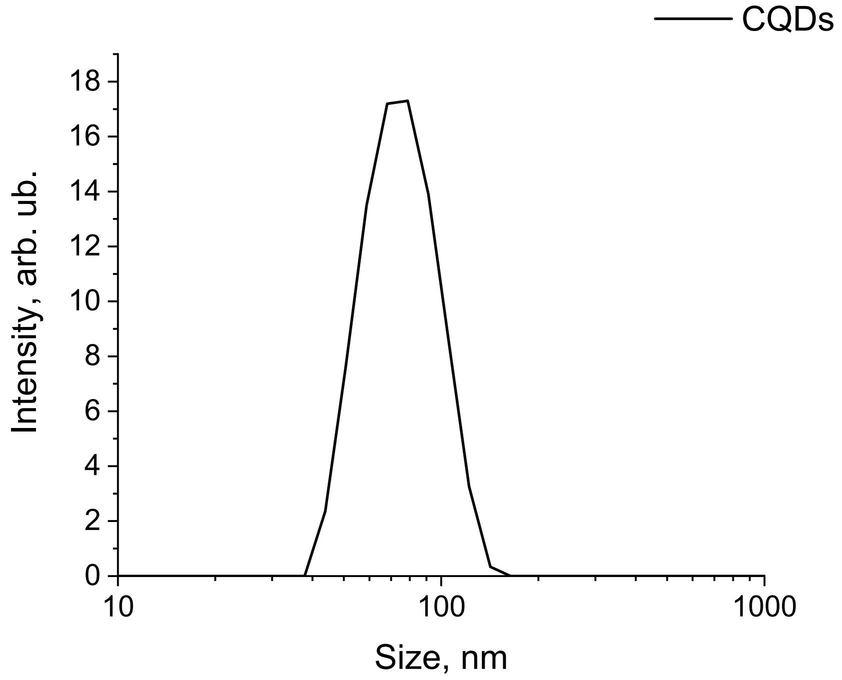

Figure S5. CQDs size distribution in aqueous suspension obtained using the DLS method.

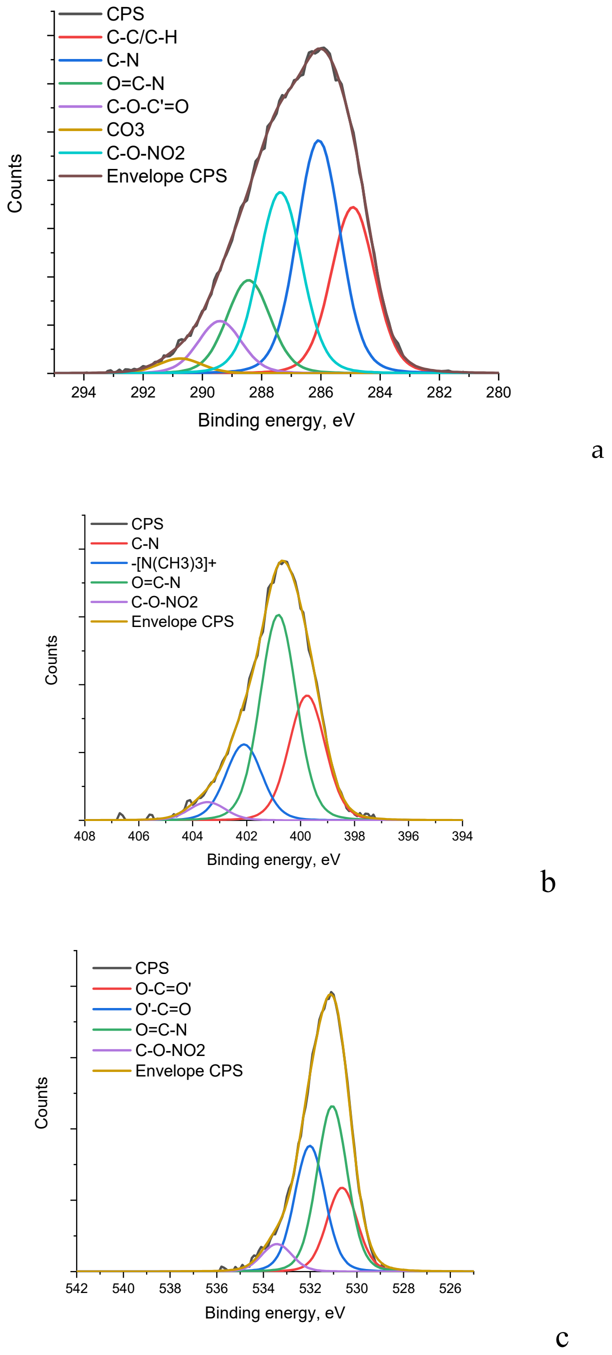

Figure S6. High resolution C1s (a), N1s (b) and O1s (c) XPS spectra of CQD.

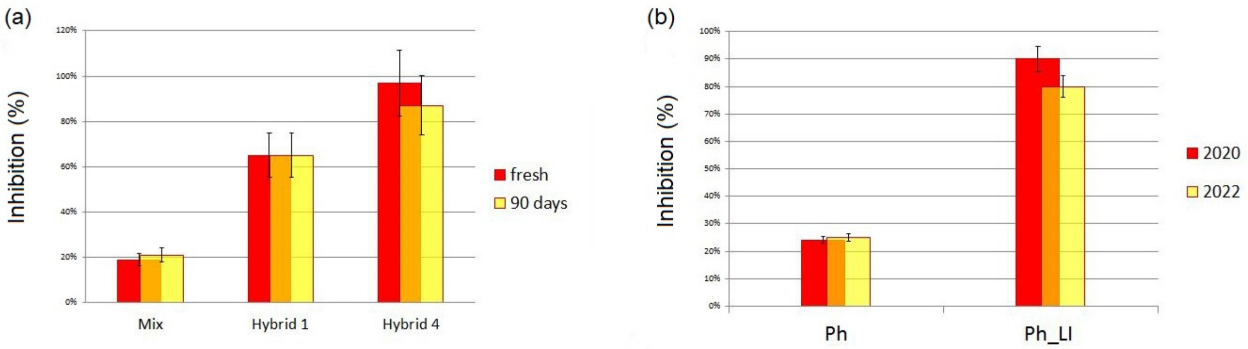

Figure S7. BChE inhibition value for (a) Mix, Hybrid1 and Hybrid4 and (b) PhAM before and after laser irradiation for prolonged period.

Table S1. Fitting parameters of decay kinetic curves for CQD, Mix, unirradiated and irradiated Hybrid1 and Hybrid4.

**Sample**	**A_1**	**τ_1 (ns)**	**A_2**	**τ_2 (ns)**	**τ_{av} (ns)**	**τ (ns)**
**CQD**	-	-	-	-	-	**9.96**
**Mix**	-	-	-	-	-	**9.66**
**Hybrid1**	-	-	-	-	-	**9.61**
**Hybrid4**	-	-	-	-	-	**9.66**
**Hybrid1_LI**	0.00466	2.01	0.00248	10.61	8.35	**8.35**
**Hybrid4_LI**	0.00619	2.27	0.00185	9.14	6.03	**6.03**

Table S2. Fitting parameters of decay kinetic curves for unirradiated and irradiated Hybrid4 on the chicken breast.

**Sample**	**A_1**	**τ_1 (ns)**	**A_2**	**τ_2 (ns)**	**τ_{av} (ns)**	**τ (ns)**
**Hybrid4**	-	-	-	-	-	**9.48**
**Hybrid4_LI**	0.00508	2.24	0.00155	9.34	6.22	**6.22**
